# Comparative analysis of blood routine, C-reactive protein, and biochemical markers in children with *Mycoplasma pneumoniae* pneumonia and its coinfections

**DOI:** 10.3389/fped.2025.1661684

**Published:** 2025-11-24

**Authors:** Xingjia Tang, Chongfeng Chen

**Affiliations:** Department of Paediatrics,The First Affiliated Hospital of Jinan University, Guangzhou, China

**Keywords:** *Mycoplasma pneumoniae*, *Haemophilus influenzae*, influenza virus, blood routine, pediatric pneumonia

## Abstract

**Introduction:**

*Mycoplasma pneumoniae* pneumonia (MPP) is a common cause of pediatric community-acquired pneumonia, and coinfections with *Haemophilus influenzae* (Hi) or influenza virus may alter disease severity. Identifying distinct laboratory patterns may help clinicians recognise coinfections early.

**Methods:**

A retrospective analysis was conducted on 140 hospitalized children with confirmed MPP (2014–2024). Patients were grouped as MPP alone (*n* = 64), MPP+Hi (*n* = 36), and MPP+influenza (*n* = 40). Clinical characteristics, complete blood count (CBC), hypersensitive C-reactive protein (hs-CRP), and biochemical indicators (ALT, AST, CK, CK-MB, urea, creatinine) were compared among groups. Correlation analyses were performed for biochemical markers.

**Results:**

Children with MPP+Hi showed the highest hs-CRP levels (23.93 ± 21.26 mg/L), longest fever duration, and longest hospital stay. The MPP+influenza group had significantly lower WBC (7.25 ± 3.50 × 10^9^/L) and platelet counts (266.00 ± 97.46 × 10^9^/L), and a higher monocyte percentage (10.18 ± 3.29%). Simple MPP cases had the highest lymphocyte percentage. No group differences were found in ALT, AST, CK, CK-MB, urea, or creatinine, although CK-MB was elevated across all groups. Correlation analysis showed weak but significant associations among AST, ALT, CK, and CK-MB.

**Conclusion:**

Coinfection type influenced inflammatory and haematological patterns in children with MPP: Hi coinfection produced stronger inflammatory responses, while influenza coinfection showed viral-associated lymphopenia and thrombocytopenia. Routine laboratory parameters may support earlier recognition of coinfections and guide more targeted clinical management.

## 1 Introduction

Pneumonia remains one of the leading causes of childhood morbidity and mortality worldwide, with *Mycoplasma pneumoniae* (MP) recognised as a major contributor to community-acquired pneumonia (CAP) in preschool and school-aged children, accounting for 10%–40% of cases (1, 2). Although *M. pneumoniae* pneumonia (MPP) is often self-limiting, some children develop severe or refractory forms characterised by prolonged fever, worsening radiographic changes, and, in rare cases, life-threatening complications (3).

In recent years, the incidence of mixed respiratory infections has risen substantially, with coinfections involving MP and other bacterial or viral pathogens increasingly reported in pediatric CAP (4, 5). Among these, Haemophilus influenzae (Hi) and influenza viruses are particularly common and clinically significant (5, 6). These pathogens, independently capable of causing significant respiratory disease, often exacerbate the clinical course when present alongside MP, leading to increased disease severity, prolonged recovery times, and greater healthcare utilization (5, 7). For instance, children with MPP and viral coinfections have been observed to experience longer fevers, more severe pneumonia, and a poorer response to standard treatments (5, 7–9).

Laboratory investigations, especially complete blood counts, hypersensitive C-reactive protein (hs-CRP), and selected biochemical markers, play a pivotal role in differentiating bacterial and viral infections (10). Elevated CRP levels often reflect bacterial involvement, while variations in white blood cell subsets and platelet counts may help to distinguish viral contributions (11, 12). Several studies have emphasized the utility of these parameters for the early recognition of mixed infections, which may allow clinicians to tailor therapy more effectively (13–15).

Despite the clinical significance, there is a paucity of studies focusing on the comparative analysis of clinical features and laboratory findings in children with MPP alone vs. those with coinfections (8). Current literature is often fragmented, focusing on individual pathogens rather than evaluating the combined impact of coinfections on disease presentation, laboratory parameters, and outcomes (16). Addressing this gap, the present study retrospectively analyzes pediatric cases of MPP, MPP + Hi, and MPP + influenza virus coinfection to identify distinctive patterns in clinical features, inflammatory markers, and biochemical indicators. By clarifying these differences, our aim is to provide clinicians with practical insights that may support earlier recognition of coinfections and more informed treatment decisions in children with pneumonia. This study adds novelty by analysing a decade-long, consistently tested paediatric cohort (2014–2024) to directly compare bacterial (*H. influenzae*) and viral (influenza) coinfections in MPP, using uniform diagnostic methods and integrated correlation analysis to reveal distinct inflammatory profiles.

## 2 Materials and methods

### 2.1 Study design and participants

This retrospective study analyzed 140 pediatric patients diagnosed with pneumonia and were admitted to the Department of Pediatrics at the First Affiliated Hospital of Jinan University between January 2014 and January 2024. The inclusion criteria were: (1) diagnosis of MPP based on the “Consensus on the Diagnosis and Treatment of Mycoplasma Pneumoniae Pneumonia (2015 Edition)"issued by the Respiratory Group of the Chinese Pediatric Society, Chinese Medical Association (2015); (2) no prior antibiotic treatment or ineffective antibiotic treatment before hospitalization; (3) completion of sputum culture, MP antibody detection, respiratory virus antigen testing, complete blood count (CBC), hypersensitive C-reactive protein (hs-CRP), and biochemical tests within 48 h of admission; and (4) confirmed detection of MP, Hi, or influenza virus antigens or nucleic acids.

Exclusion criteria included: positive identification of other respiratory viruses or bacteria (besides Hi), positive sputum culture for bacteria other than Hi, history of chronic underlying diseases (e.g., congenital heart disease, immunodeficiencies, bronchopulmonary dysplasia), and inclusion of neonatal patients. We focused specifically on *H. influenzae* and influenza virus because these were the most frequent coinfecting agents detected in our cohort over the study period. Other bacterial and viral pathogens were excluded due to their low prevalence, which would have yielded very small subgroups and limited statistical comparability. Restricting the analysis to Hi and influenza virus enabled clearer identification of clinically relevant differences. The patients were divided into three groups based on their infection status: Group A (MPP alone, *n* = 64), Group B (MPP with Hi coinfection, *n* = 36), and Group C (MPP with influenza virus coinfection, *n* = 40).

### 2.2 Microbiological and virological testing

Deep respiratory tract secretions were obtained using sterile suction catheters. Sample quality was assessed via Gram stain microscopy, requiring >25 white blood cells and <10 epithelial cells per high-power field for specimen acceptance. Qualified sputum samples were inoculated onto chocolate agar supplemented with bacitracin and incubated at 35–37°C in a 5% CO₂-enriched atmosphere for 24–48 h. *Haemophilus influenzae* colonies were identified based on characteristic morphology, positive oxidase test, and growth dependence on both X (hemin) and V (nicotinamide adenine dinucleotide) factors. Confirmatory biochemical identification was performed using standard techniques.

*Mycoplasma pneumoniae* infection was confirmed by two methods: (1) detection of MP-IgM antibodies via the SERODIA-MYCO II particle agglutination test, with a positive result defined as a titer ≥1:160; and (2) detection of MP RNA from throat swabs using RNA isothermal amplification combined with a gold nanoparticle-based lateral flow assay using commercially available kit (Wuhan Zhongzhi Biotechnology Co., Ltd.). RNA extraction was performed according to the kit instructions. Internal positive and negative controls were included in each run.

For respiratory virus detection, nasopharyngeal swabs were analyzed for six common viruses: respiratory syncytial virus, adenovirus, human metapneumovirus, and parainfluenza virus types 1–3. Detection was performed via one-step reverse transcription polymerase chain reaction (RT-PCR) using commercial kits (Shanghai Bojie Medical Science & Technology Co., Ltd.), following the manufacturer's protocols. Only patients with positive detection of influenza virus antigens or nucleic acids were included.

All laboratory tests and diagnostic workflows were performed using the same core platforms and quality-control procedures throughout 2014–2024. CBC analyses were conducted on the Sysmex XN-Series, hs-CRP on the BN ProSpec® (Siemens Healthineers), and biochemical assays on the OLYMPUS AU640 analyzer. The same Mycoplasma pneumoniae antibody (SERODIA-MYCO II) and RNA detection kits (Wuhan Zhongzhi Biotechnology Co., Ltd.) were used across the study period. Routine internal calibration and participation in external quality-assurance programs ensured analytical consistency despite the long retrospective timeframe.

### 2.3 Laboratory investigations

#### 2.3.1 Complete blood count (CBC)

CBC parameters, including white blood cell count (WBC), neutrophil percentage (N%), lymphocyte percentage (L%), monocyte percentage (M%), eosinophil percentage (EO%), basophil percentage (BASO%), absolute counts for each cell type (N#, L#, M#, EO#, BASO#), platelet count (PLT), mean platelet volume (MPV), mean corpuscular volume (MCV), mean corpuscular hemoglobin (MCH), mean corpuscular hemoglobin concentration (MCHC), red cell distribution width-coefficient of variation (RDW-CV), and red cell distribution width-standard deviation (RDW-SD), were measured using an automated hematology analyzer (Sysmex XN-Series, Sysmex Corporation, Kobe, Japan). Routine internal and external quality control procedures were followed in accordance with hospital and national laboratory standards.

#### 2.3.2 Hypersensitive C-reactive protein (hs-CRP)

Hs-CRP levels were measured using an immunoturbidimetric assay on an automatic specific protein analyzer (BN ProSpec®, Siemens Healthineers, Germany). A positive threshold was defined as ≥0.50 mg/L. Calibration and quality controls were performed daily according to the manufacturer's guidelines.

#### 2.3.3 Biochemical tests

Fasting venous blood samples were analyzed using an OLYMPUS AU640 automatic biochemical analyzer (Olympus Corporation, Tokyo, Japan). Parameters included alanine aminotransferase (ALT) and aspartate aminotransferase (AST) measured by the International Federation of Clinical Chemistry recommended enzymatic rate method without pyridoxal phosphate activation; creatine kinase (CK) and creatine kinase-MB (CK-MB) measured by the enzymatic method; urea measured by the urease–glutamate dehydrogenase method; and creatinine (CREA) measured by the enzymatic method. Quality control materials (two levels) were run daily to ensure accuracy and precision.

All patients underwent these laboratory tests within 48 h of admission.

### 2.4 Statistical analysis

Data were analysed using SPSS Statistics version 29.0 (IBM Corp., Armonk, NY, USA). The Kolmogorov–Smirnov test was used to assess the normality of data. Normally distributed continuous variables were expressed as mean ± standard deviation and compared between groups using independent-sample *t*-tests or one-way analysis of variance (ANOVA), followed by Tukey's HSD test. Non-normally distributed data were presented as medians (P25, P75) and compared using the Kruskal–Wallis test, followed by Dunn's multiple range test. Categorical variables were expressed as percentages and compared between groups using the Chi-square (*χ*^2^) test. No formal correction for multiple comparisons was applied, as this was an exploratory study. Over-correction could increase Type II error and obscure potential biological patterns; therefore, *p*-values are presented descriptively. Correlation analyses were conducted using Pearson's or Spearman's methods, depending on data distribution. *P*-value < 0.05 was considered statistically significant. Effect sizes for significant ANOVA results were small (partial *η*^2^ < 0.1), indicating modest group differences.

## 3 Results

### 3.1 General clinical characteristics

Significant differences were observed in age, cough duration, fever duration before admission, and length of hospital stay among the three groups (*P* < 0.05) (Table 1). Children in Group B (MPP with Hi) were the youngest (4.08 ± 2.66 years), followed by Group A (4.91 ± 1.61 years), while Group C (MPP with influenza virus) included older children (5.15 ± 2.95 years). The duration of coughing before admission was longest in Group A (15.26 ± 12.71 days), significantly longer than in Groups B (8.45 ± 9.07 days) and C (7.46 ± 6.72 days). Fever duration was longest in Group B (5.09 ± 4.85 days), compared to Group C (3.58 ± 3.25 days) and Group A (2.50 ± 2.61 days). Hospital stays were also significantly longer in Group B (10.11 ± 4.81 days) compared to Groups A (8.11 ± 2.44 days) and C (8.05 ± 2.04 days).

**Table 1:** Comparison of age, symptom duration, and hospital stay among children with Mycoplasma pneumoniae pneumonia, with and without coinfections (children with MPP + H. influenzae were youngest with longest fever and hospital stay; MPP-only had longest cough).

Group	Age (years)	Cough duration (days)	Fever duration (days)	Hospital stay (days)
A (MPP only)	4.91 ± 1.6^a^	15.26 ± 12.7^a^	2.50 ± 2.6^c^	8.11 ± 2.4^b^
B (MPP + Hi)	4.08 ± 2.6^b^	8.45 ± 9.0^b^	5.09 ± 4.8^a^	10.11 ± 4.8^a^
C (MPP + Influenza)	5.15 ± 2.9^a^	7.46 ± 6.7^b^	3.58 ± 3.2^b^	8.05 ± 2.0^b^
F-value	37.13	30.51	49.63	43.71
*p*-value	0.013	<0.001	<0.001	<0.001

Values are mean ± standard deviation. Different superscript letters within the same column indicate statistically significant differences between groups (*p* < 0.05).MPP, *Mycoplasma pneumoniae* pneumonia; Hi, *Haemophilus influenzae*.

### 3.2 Complete blood count and hs-CRP

Significant differences were observed among the three groups in hs-CRP levels, WBC count, neutrophil count (N#), lymphocyte count (L#), monocyte count (M#), platelet count (PLT), lymphocyte percentage (L%), and monocyte percentage (M%). Group B exhibited the highest hs-CRP levels (23.93 ± 21.26 mg/L a), significantly higher than Groups A (12.80 ± 24.01 mg/L ab) and C (9.31 ± 11.87 mg/L b) (*P* = 0.006). WBC counts were significantly lower in Group C (7.25 ± 3.50 × 10⁹/L b) compared to Groups A (10.79 ± 5.10 × 10⁹/L a) and B (12.04 ± 5.85 × 10⁹/L a) (*P* < 0.001). Similarly, neutrophil counts (N#) and lymphocyte counts (L#) were lowest in Group C (*P* = 0.003 and *P* = 0.001, respectively). Monocyte counts (M#) were highest in Group B and lowest in Group C (*P* = 0.006). Platelet counts were significantly lower in Group C (*P* = 0.014). Lymphocyte percentage (L%) was highest in Group A and markedly lower in Groups B and C (*P* = 0.045). Monocyte percentage (M%) was highest in Group C compared to Groups A and B (*P* = 0.010). No statistically significant differences were found among groups in neutrophil percentage (N%), eosinophil counts (EO#), or basophil counts (BASO#) (*P* > 0.05) (Table 2). Similarly, no statistically significant differences were found among the three groups in terms of red blood cell indices and platelet parameters (Table 3).

**Table 2:** Comparison of inflammatory markers and white blood cell counts among children with Mycoplasma pneumoniae pneumonia, with and without coinfections (hs-CRP highest in MPP + H. influenzae; WBC and platelets lowest in MPP + influenza).

Parameter	Group A (MPP)	Group B (MPP + Hi)	Group C (MPP + Influenza)	Reference value	F-value	*p*-value
HsCRP (mg/L)	12.80 ± 24.0^ab^	23.93 ± 21.2^a^	9.31 ± 11.8^b^	0–8	5.29	0.006
WBC (×10⁹/L)	10.79 ± 5.1^a^	12.04 ± 5.8^a^	7.25 ± 3.5^b^	4.3–11.3	10.14	<0.001
N# (×10⁹/L)	6.06 ± 4.4^ab^	7.65 ± 4.4^a^	4.22 ± 2.9^b^	1.6–7.8	6.00	0.003
L# (×10⁹/L)	3.65 ± 1.9^a^	3.22 ± 2.0^ab^	2.21 ± 1.4^b^	1.5–4.6	7.53	0.001
M# (×10⁹/L)	0.84 ± 0.4^ab^	1.03 ± 0.4^a^	0.72 ± 0.3^b^	0.13–0.76	5.34	0.006
PLT (×10⁹/L)	300.84 ± 108.0^ab^	353.08 ± 105.8^a^	266.00 ± 97.4^b^	167–453	4.43	0.014
N%	51.93 ± 18.8^a^	60.51 ± 17.8^a^	56.09 ± 19.1^a^	31–70	2.49	>0.05
L%	39.64 ± 18.1^a^	29.07 ± 15.0^b^	30.34 ± 17.8^b^	23–59	3.17	0.045
M%	8.23 ± 2.8^ab^	9.03 ± 3.5^ab^	10.18 ± 3.2^a^	2–11	4.78	0.01

Values are presented as mean ± standard deviation. Different letters following the means indicate statistically significant differences between groups (*p* < 0.05). MPP, Mycoplasma pneumoniae pneumonia; Hi, Haemophilus influenzae; HsCRP, hypersensitive C-reactive protein; WBC, white blood cell count; N#, neutrophil count; L#, lymphocyte count; M#, monocyte count; PLT, platelet count; N%, neutrophil percentage; L%, lymphocyte percentage; M%, monocyte percentage.

**Table 3:** Comparison of red blood cell indices and platelet parameters among children with Mycoplasma pneumoniae pneumonia, with and without coinfections (No significant differences in red-cell or platelet indices among groups).

Parameter	Group A (MPP)	Group B (MPP + Hi)	Group C (MPP + Influenza)	Reference value	F-value	*p*-value
EO# (×10⁹/L)	0.12 [0.2]	0.09 [0.2]	0.06 [0.1]	0–0.68	2.51	>0.05
BASO# (×10⁹/L)	0.02 [0.0]	0.02 [0.0]	0.02 [0.0]	0–0.07	0.32	>0.05
EO% (%)	1.31[2.4]	0.67 [1.6]	0.78 [2.1]	0–9	1.42	>0.05
BASO% (%)	0.27 [0.3]	0.15 [0.3]	0.19 [0.6]	0–1	1.99	>0.05
MCV (fL)	80.39 ± 5.1	81.63 ± 5.5	81.78 ± 6.03	77–92	0.64	>0.05
MCH (pg)	26.66 ± 2.0	26.90 ± 2.2	27.02 ± 2.28	25–34	0.16	>0.05
MCHC (g/L)	331.63 ± 8.1	329.18 ± 10.2	330.01 ± 8.00	310–355	1.01	>0.05
MPV (fL)	8.75 ± 0.9	8.69 ± 1.2	8.55 ± 1.05	8–12.5	0.46	>0.05
RDW-CV (%)	12.90 ± 1.7	13.31 ± 1.8	13.35 ± 1.47	11.5–15	1.10	>0.05
RDW-SD (fL)	37.99 ± 2.9	39.24 ± 3.8	38.40 ± 2.08	–	2.00	>0.05

Values are mean ± standard deviation or median [P25, P75], as appropriate. MPP, *Mycoplasma pneumoniae* pneumonia; Hi, *Haemophilus influenzae*; EO#, eosinophil count; BASO#, basophil count; EO%, eosinophil percentage; BASO%, basophil percentage; MCV, mean corpuscular volume; MCH, mean corpuscular hemoglobin; MCHC, mean corpuscular hemoglobin concentration; MPV, mean platelet volume; RDW-CV, red cell distribution width–coefficient of variation; RDW-SD, red cell distribution width–standard deviation.

### 3.3 Complete blood count and hs-CRP

No significant differences were found among the groups for ALT, AST, CK, CK-MB, UREA, and CREA levels (*P* > 0.05) (Table 3). However, CK-MB values in all groups exceeded the normal reference range, suggesting myocardial involvement despite the absence of statistically significant intergroup differences (Table 4).

**Table 4:** Comparison of biochemical indicators among children with *Mycoplasma pneumoniae* pneumonia, with and without coinfections (No group differences in biochemistry; CK-MB elevated across all groups).

Parameter	Group A (MPP)	Group B (MPP + Hi)	Group C (MPP + Influenza)	Reference value	*F*-value	*p*-value
ALT(U/L)	17.61 ± 7.9	20.72 ± 33.0	17.20 ± 7.2	7–40	0.47	>0.05
AST (U/L)	40.17 ± 10.0	42.69 ± 31.9	44.00 ± 17.7	13–35	0.49	>0.05
CK (U/L)	153.44 ± 70.8	149.69 ± 78.9	146.35 ± 98.8	50–310	0.09	>0.05
CK-MB(U/L)	42.44 ± 21.1	38.36 ± 28.6	35.53 ± 23.0	0–24	1.21	>0.05
UREA (mmol/L)	3.85 ± 1.4	3.50 ± 1.3	3.62 ± 0.8	1.5–7.5	0.98	>0.05
CREA (*μ*mol/L)	33.08 ± 9.2	33.35 ± 9.0	35.63 ± 10.0	41–111	0.97	>0.05

Values are presented as mean ± standard deviation. MPP, *Mycoplasma pneumoniae* pneumonia; Hi, *Haemophilus influenzae*; ALT, alanine aminotransferase; AST, aspartate aminotransferase; CK, creatine kinase; CK-MB, creatine kinase-MB isoenzyme; UREA, blood urea nitrogen; CREA, creatinine.

**Figure 1:**
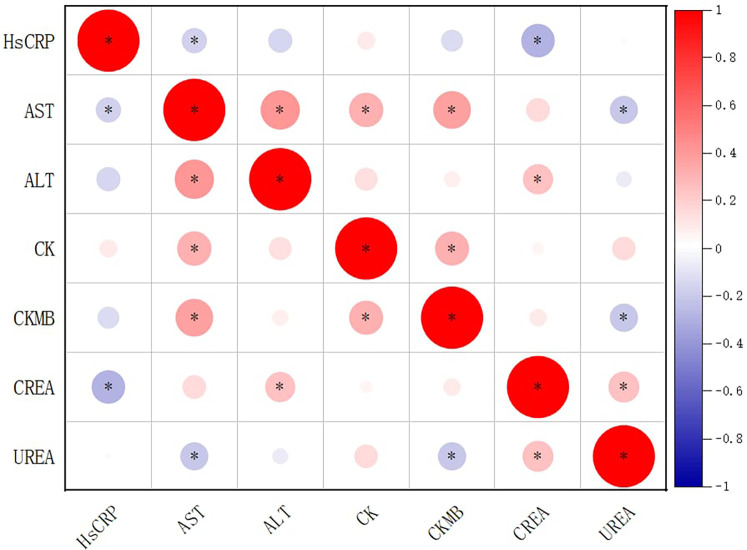
Correlation matrix of biochemical indicators in children with *Mycoplasma pneumoniae* pneumonia and coinfections. Circle size and color represent correlation strength and direction (red = positive; blue = negative). Asterisks (*) denote correlations significant at *p* < 0.05. Based on reported *p*-values, correlations between AST and ALT, AST and CK, AST and CK-MB, CK-MB and CK, and CK-MB and AST would remain significant after multiple-testing correction. Diagonal cells represent self-correlations (r = 1) and are retained for completeness, although they are mathematically trivial.

Correlation analysis among biochemical markers showed that AST was positively correlated with ALT (r = 0.402, *P* < 0.001), CK (r = 0.308, *P* < 0.001), and CK-MB (r = 0.387, *P* < 0.001), and negatively correlated with UREA (r = −0.206, *P* = 0.015) and hs-CRP (r = −0.169, *P* = 0.046). CK-MB was positively correlated with CK (r = 0.302, *P* < 0.001) and AST (r = 0.387, *P* < 0.001), and negatively correlated with UREA (r = −0.210, *P* = 0.013). Both types of correlation was very weak. No significant correlations were found between CK-MB and ALT, CREA, or hs-CRP (Figure 1).

## 4 Discussion

*Mycoplasma pneumoniae* pneumonia is increasingly reported alongside coinfections with Hi or influenza virus, which can worsen clinical outcomes in children due to their immature immunity and physiological vulnerability (3, 17). In this study, we compared the clinical and laboratory characteristics of children with MPP alone and those with coinfections involving Hi or influenza virus. We found that Hi coinfection was associated with elevated inflammatory markers, prolonged fever, and longer hospital stays, whereas influenza virus coinfection was characterized by lymphopenia and thrombocytopenia, reflecting differing host immune responses to bacterial vs. viral pathogens (18–20). These patterns are consistent with prior reports showing that Hi coinfection amplifies inflammatory responses through innate immune activation, resulting in higher hs-CRP and neutrophil counts (21–23), while influenza virus coinfection impairs host defences by inducing lymphocyte apoptosis and immune suppression (24). Clinically, the observed patterns suggest therapeutic implications. Children coinfected with *Hi* showed the highest hs-CRP, prolonged fever, and hospital stays, indicating a stronger inflammatory response that may warrant early antibacterial treatment. In contrast, MPP + influenza virus cases showed lymphopenia and thrombocytopenia, supporting timely antiviral use. Elevated CK-MB across all groups highlights the need for cardiac monitoring in pediatric MPP regardless of coinfection.

Consistent with previous studies, age and symptom duration also varied across groups: Hi coinfections were more common in younger children, whereas influenza coinfections occurred more often in preschool and school-aged children, reflecting age-related susceptibility patterns (12, 25–28). These trends are consistent with epidemiological data and reinforce the need for age-tailored clinical vigilance. Previous research has reported that coinfections can worsen MPP severity (8, 24, 29). Choo et al. reported that respiratory viral coinfections correlated with longer fever duration and increased risk of severe pneumonia (30). These findings align with our study and extend prior work by directly comparing both bacterial (Hi) and viral (influenza virus) coinfections in MPP.

In addition, platelet counts were significantly lower in the MPP + influenza group, supporting the immunosuppressive effect of viral coinfection. Reduced platelet levels are increasingly recognized as a marker of severity in respiratory infections (24, 31). Although total WBC counts did not differ significantly between MPP and MPP + Hi groups, this may reflect sampling limitations or undetected co-pathogens (32). We also observed weak correlations among biochemical markers such as AST, ALT, CK, and CK-MB. Although these relationships were statistically significant, the effect sizes were small (r ≈ 0.3–0.4) and most likely reflect shared metabolic responses, such as concurrent hepatocellular and myocellular enzyme release, rather than clinically meaningful associations. While this overlap is expected given the enzymes’ related tissue origins, the consistent elevation of CK-MB across all groups remains noteworthy and may indicate mild, nonspecific myocardial or skeletal muscle involvement, warranting cautious clinical observation rather than a presumption of cardiac injury. Such CK-MB elevation in pneumonia may also arise from systemic inflammation, hypoxia, or skeletal muscle strain rather than direct myocardial damage, and therefore should be interpreted with caution (33–35).

From a practical perspective, these observations suggest that routine laboratory data may provide early, actionable clues while awaiting pathogen confirmation. For example, elevated hs-CRP with prolonged fever may alert clinicians to possible Hi coinfection and support timely antibacterial escalation, whereas lymphopenia and thrombocytopenia should raise suspicion of influenza virus coinfection and prompt early antiviral consideration or closer monitoring. Elevated CK-MB, seen across all groups, underscores the importance of cardiac monitoring in children with MPP. Importantly, we are not proposing a diagnostic score. Rather, our findings indicate that widely available laboratory parameters can serve as complementary, rapid indicators, particularly valuable in settings where multiplex diagnostic panels are unavailable, delayed, or cost-prohibitive.

This study has several limitations. It was retrospective and conducted in a single center, limiting generalizability. Subgroup sizes were modest, preventing the use of robust multivariate analyses and restricting our findings to exploratory associations. We also focused only on Hi and influenza virus, as these were the predominant coinfections in our cohort; results may not apply to less common pathogens. In addition, potential seasonal variation in infection rates was not analyzed due to lack of detailed temporal data. Future multicenter studies with larger sample sizes are needed to validate these findings and to assess whether laboratory parameters could be integrated into predictive algorithms alongside molecular diagnostics.

In summary, our results highlight distinct clinical and laboratory profiles of MPP coinfections. Hi coinfection was linked to heightened inflammatory responses and longer illness, while influenza virus coinfection was associated with immune suppression and thrombocytopenia. These patterns may support earlier recognition of coinfections and more tailored treatment, serving as an adjunct to, but not a replacement for, pathogen-directed diagnostic testing.

## Data Availability

The original contributions presented in the study are included in the article/Supplementary Material, further inquiries can be directed to the corresponding author.
